# Experimental Investigation and CFD Analysis of Pressure Drop in an ORC Boiler for a WHRS Implementation

**DOI:** 10.3390/s22239437

**Published:** 2022-12-02

**Authors:** Concepción Paz, Eduardo Suárez, Adrián Cabarcos, Antonio Díaz

**Affiliations:** CINTECX, Universidade de Vigo, Campus Universitario Lagoas-Marcosende, 36310 Vigo, Spain

**Keywords:** ORC, WHRS, CFD, pressure drop, boiler, multiphase flow, mini/micro channels

## Abstract

Waste heat dissipated in the exhaust system of a combustion engine represents a major source of energy to be recovered and converted into useful work. The Waste Heat Recovery System (WHRS) based in an Organic Rankine Cycle (ORC) is an approach for recovering energy from heat sources, achieving a significant reduction in fuel consumption and, as a result, exhaust emissions. This paper studies pressure drop in an ORC shell-and-tubes boiler for a WHRS implementation experimentally and with computational simulations based on a 1-dimensional heat transfer model coupled with 3D calculations. An experimental database is developed, using ethanol in a pressure range of 10–15 absolute bar as working fluid, with mass fluxes inside the tubes in the range of 349.31 kg/s-m^2^ and 523.97 kg/s-m^2^, and inlet temperatures in the range of 60 °C and 80 °C. Thus, the friction factor of different regions of the boiler were estimated using both CFD simulations, experimental data, and bibliographic correlations. Simulations of operating points and the results of the experimental test bench showed good agreement in pressure drop results, with a mean absolute error of 15.47%, without a significant increment in the computational cost.

## 1. Introduction

The increasing interest in emission reduction due to restrictive environmental regulations and the rise in fuel costs demands new technologies to enhance fuel efficiency in internal combustion engines. Most modern diesel engines are achieving up to about 40% in thermal efficiency. The losses are related to waste heat dissipated in several routes, such as the coolant system, convection, and radiation from the engine block and the exhaust system. In fact, this last one represents up to another 40% of the total fuel energy at maximum power point [1,2,3,4,5]. The Organic Rankine Cycle (ORC) technology is an effective method to recover energy from low-temperature waste heat that has been studied in depth in the last decades and has been implemented in a wide variety of fields in the industry, such as marine engines [6,7,8,9,10,11,12,13,14,15,16,17,18], light duty vehicles [19,20,21,22,23,24], heavy-duty vehicles [22,25,26,27,28,29,30,31,32,33,34], biodiesel engines [35], heat and power generation [36,37,38,39], geothermal energy [40,41,42], solar energy [43,44,45,46], or biogas plants [47,48]. Energy saving, efficiency improvement, and emissions reduction of WHRS based on an ORC vary depending on the application, the energy output, or the exergy efficiency [16]. Studies found in the bibliography report a maximum fuel saving between 10% and 15% [9,12,47,49,50,51].

The ORC based on vapor generation in a secondary circuit represents an indirect method of waste heat recovery (WHR). This technique has advantages compared with the so-called direct WHR techniques, such as electrical turbo compounding [52], or mechanical turbo compounding [53], which uses a power turbine fitted to the vehicle exhaust and has a much higher impact on the engine pumping losses. In addition, an ORC achieves a high waste energy conversion and it is cheaper than other WHR techniques such as thermoelectric generators [2].

Restrictions on the implementation of the technology must also be considered. On-road vehicles are more challenging in incorporating an ORC in the engine, due to their transient and highly variable operating conditions, which implies the need of implementing control systems [22]. Cost, package considerations, weight [54], additional cooling demand [55], increased back pressure [6], reliability, and safety, must also be considered.

Pressure drop is a phenomenon to be considered in heat exchangers, such as the boiler in an ORC. A small pressure drop is necessary to enable the fluids to move, but large pressure drops reduce efficiency, require using a lot of pump power, and can cause premature equipment failure. In contrast, the positive result of pressure drop is the higher turbulence obtained, which is desirable in heat exchangers since it improves heat transfer. Multiphase flow that occurs in the boiler of an ORC is quite a complex physical phenomenon since it involves heat and mass transfer between phases, and other aspects such as critical heat flux, flow instability, flow-induced vibration, etc. [56]. Prediction of pressure drop characteristics mainly depend on the understanding of thermal-hydraulic characteristics. For reliable and safe operation, optimization of the operating conditions of the boiler is necessary. Therefore, regarding design and operational tasks, it is essential to have knowledge of the local behavior of thermal-hydraulic characteristics at different operating conditions in the evaporator [57]. Several previous studies have evaluated WF pressure drop in heat exchangers in different fields of engineering, such as Cioncolini and Santini [58], Prabakaran et al. [59], Li et al. [60], Zhang and Haglind [61], or Raju et al. [62].

Regarding numerical studies of heat exchangers, the biggest challenge in the simulation of a boiler is modeling phase transition. There are many works that attempt a mathematical 1-dimensional prediction of the thermodynamics in multiphase heat exchangers and two different approaches are related in the literature: moving-boundary models that employ one domain for each phase and track the phase boundaries [63,64,65,66], and discretized models [40], that divide the path of the flow into several steps with average properties. While moving-boundary models are reported to have higher numerical efficiency due to lower model orders, discretized models promise higher accuracy and a better understanding of multiphase flow [63].

In particular, the boiler studied in the present paper uses high-aspect ratio straight corrugated tubes, so-called mini/micro channels. Mini/micro channels are widely implemented in the industry since they have the advantage of inducing a high-velocity gradient on the fluid, which enhances heat transfer due to convective diffusion, but at the cost of producing shear force and a high-pressure drop at the same time. Moreover, mini/micro channels can generate a high-pressure drop as a result of two physical phenomena associated with phase change: compressibility, due to the difference between specific volume between liquid and vapor phases; and flashing, which occurs when the liquid phase evaporates due to a drop in the pressure. Both high compressibility and flashing can lead to two-phase choking [67]. It should also be noted that multiphase pressure drop in mini/micro channels is broadly studied in the literature, where several correlations are proposed in single tube assessments [68,69,70,71,72,73,74]. However, there is a lack of studies focused on correlations for multiphase pressure drop in full heat exchangers [75].

An alternative to a numerical 1-dimensional calculation to analyze the physics of a boiler is computational fluid dynamic (CFD) simulation, where both two-dimensional and three-dimensional calculations can be performed. Different multiphase models are available in commercial software, the so-called Euler–Euler approach: the VOF (volume of fluid) model, the mixture model, and the Eulerian model [76]. All of them require modeling the complex interactions between phases, either tracking through the domain boundary between phases in immiscible fluids (VOF), solving the mixture momentum equation and calculating the relative velocities in interpenetrating continua phases (mixture model), or calculating the momentum exchange between phases (Eulerian model). The interactions described typically require using empirical models for heat transfer, bubble physics, or forces between phases, which are highly dependent on fluid flow conditions, wall heat flux, system pressure, geometry, hydraulic diameter, and wall roughness [77]. In addition, it is a well-known issue that most CFD software face convergence difficulties in multi-phase calculations, such as numerical instability in non-structured meshes, and higher computational cost. This fact is mainly due to an increase in the number of equations involved in the calculations. This limitation can be tackled in a nontrivial way by creating a mesh with an approximate grid size of the physical processes involved [78,79]. Nevertheless, the geometry of the industrial components is often excessively complex to afford a refined mesh that considers multi-phase fluid dynamics. Consequently, a multiphase CFD simulation would lead to an enormous computational effort and an increase in the calculation time, compromising or even preventing fast responses to the engineering industry.

There are not many studies in the literature that study evaporation by means of CFD simulations in heat exchangers due to the difficulties and challenges previously described. Mali et al. [57] studied pressure drop in vertical tubes for a boiler at high pressure, using the Eulerian–Eulerian model and dividing the length of the tube into different sections regarding vapor fraction. Wang et al. [80] and Shi et al. [81,82] used de RPI wall boiling model proposed by Kurul and Podowski [83] to simulate a particular region of an evaporator, taking advantage of the periodic distribution of the tubes inside the shell to predict the thermal-hydraulic characteristics of the tube. Mohammed et al. [84] used the VOF model to simulate the evaporation and condensation of the acetone in a horizontal circular tube. Cappelli et al. [85] used the VOF model to investigate gas flow rate, gas temperature, and liquid flow rate on mass transfer in a bubble column evaporator. Höhne [86] used the homogeneous multiphase model to simulate in a CFD software the heat and mass transfer process and compared the results with experiments from the literature. The studies reviewed have in common that neither specifically evaluate WF pressure drop via CFD, and they simulate simple geometries consisting in one straight tube or a specific region of the heat exchanger, and they are focused on studying mass transfer or thermal properties.

For all the above, there is an increasing interest in the study of pressure drop in multiphase flow in heat exchangers and the challenges involved in CFD simulations of multiphase flow in complex geometries. This paper presents a methodology to predict multiphase pressure drop in shell and tube heat exchangers by means of CFD simulation and our own experimental data. The limitations of CFD in the field of multiphase flow are addressed using a well-contrasted tool to couple a 1-dimensional model to a CFD software, presented by the authors of the present paper previously in 2019 [87]. The correlations to predict pressure drop used in the 1-dimensional model are based on bibliographic references for single-phase flow and multiphase flow, the experimental results obtained in our own experimental test bench, and full CFD simulations are used to model the local pressure drop factor of the boiler studied.

The present paper aims to study one of the main components of the ORC, the boiler, both experimentally and numerically. This work continues the studies published in a previous paper by the authors [87] where a 1-dimensional model was coupled to a computational fluid dynamics (CFD) commercial software in a boiler of an ORC in a WHRS. This previous paper focused on understanding heat transfer experimentally in a test bench via infrared thermography and using CFD software. However, the present paper is focused on analyzing and modeling the pressure drop of the multiphase flow in the WF in a boiler for an ORC by means of CFD simulations and experimental results, and proposing a methodology to estimate the local pressure drop factor in single-phase and multiphase flow.

In Section 2 of this paper, the test bench setup and the boiler of the study are presented. In Section 3, the experimental results are shown. Section 4 describes the meshing process. Section 5 explains the numerical approach. Section 6 presents the numerical and experimental methodology to obtain the friction factor and local pressure drop factor in the single-phase and multiphase stages of the WF inside the boiler. The CFD results are discussed and contrasted with experimental data in Section 7. Finally, conclusions regarding the methodology presented in this study are recapitulated in Section 8.

## 2. Experimental Setup

**Figure 1:**
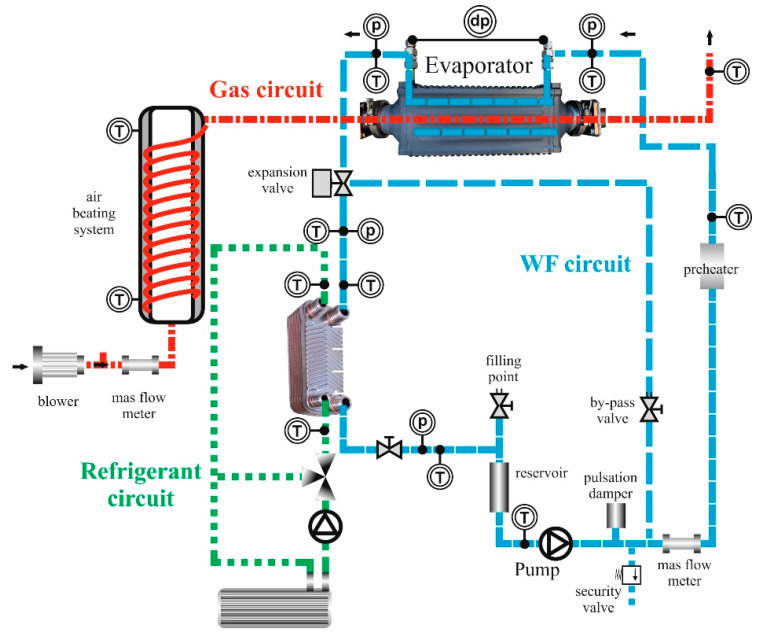
Schematic diagram of the test bench. The gas, WF, and water refrigerant circuits are represented in red, blue, and green, respectively. (T) Denotes temperature sensors, (p) absolute pressure sensors, and (dp) differential pressure sensors.

Figure 1 depicts the experimental test bench used in the present work, which allows analysis of the boilers and evaporators in a broad range of mass flows, temperatures, and pressures. The test bench has three main circuits: air, ethanol, and cooling water. The ethanol accumulated in the tank is pumped and preheated before its entrance to the evaporator, where it receives heat from hot air, previously heated with an electric resistor. Subsequently, ethanol is cooled down in the condenser by transferring its heat to a cooling water circuit.

There are multiple temperature and pressure sensors in several points of interest in the test bench. Thus, temperatures, pressures, and mass flows can be measured. These points of interest include the evaporator inlet and outlet, and the ethanol pre-heater inlet and outlet, among others. K-type thermocouples were disposed for the gas side temperature measurement with an uncertainty of 1.5 °C, while T-type thermocouples were disposed for the WF and the metal sides with an uncertainty of 0.5 °C. The sensors used for pressure drop measurements were absolute pressure transmitters, which featured a single crystal silicon resonant sensor, with an accuracy span of ±0.055%, and a differential pressure sensor, which consists of a piezo-resistance silicon-type sensor with an accuracy of 0.25%.

**Figure 2:**
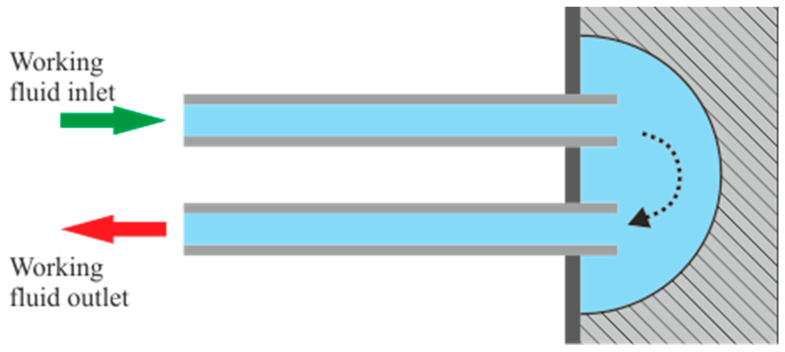
Schematic representation of a mixing chamber linking one row of tubes to the following one. The arrows represent the direction of the WF flow, colored in blue.

The boiler used to validate the model is a flow shell and corrugated-tube heat exchanger in crossed flow and counter flow, made entirely of steel AISI 316l, with the exception of the tubes, made of steel AISI 304. Air flows perpendicularly through a bundle of staggered tubes, while the WF flows inside those tubes. After the WF intake, WF mass flow is divided in rows of 14 tubes that converge in a mixing chamber at opposite sides of the boiler. In that mixing chamber, the WF converges and is also divided in another row of 14 tubes. This pattern is repeated in a total of 50 steps of rows of tubes inside the shell of the boiler, accounting for a total of 700 corrugated tubes of 5 mm of diameter and 0.25 mm of wall thickness. After the 50th row of tubes, the WF mass flow converges in the WF outtake and leaves the boiler into the WF circuit of the test bench. A schematic representation of a mixing chamber is shown in Figure 2, where each of the tubes displayed represents all the 14 tubes belonging to the same row.

## 3. Experimental Results

Two sets of experiments were performed: one with very low heat exchange, with air below the saturation temperature to ensure that the WF remains as liquid and to analyze pressure drop in the monophasic stage. The other experiment tested the operating conditions of the boiler to analyze the pressure drop in the multiphase stage. In every case, the fluids involved were ethanol as WF flowing inside the tubes and dry air at atmospheric pressure flowing outside the tubes and inside the shell of the boiler in perpendicular direction to the tubes.

The first set of experiments used to study monophasic pressure drop are listed in Table 1. Note that all the inputs in the test bench are fixed, except mass flow of the WF.

The second set of experiments was performed in operating conditions, where the WF enters the boiler as subcooled liquid and exists as an overheated vapor. Multiphase experiments were studied in several rounds of experiments, where WF inlet conditions were fixed and dry air conditions started at 500 °C and 50 kg/h and were gradually increased 10 °C and 1 kg/s every 20 min and at every measurement of the pressure drop. Outlet temperature was monitored and, if after 20 min the outlet temperature did not surpass the saturation temperature and provided the experiment had already reached a steady stage, the experimental point was dismissed and the inlet air temperature and inlet air mass flow were increased again. Only when the outlet temperature sensor showed a measurement significantly higher than the saturation temperature was it assumed that the WF had reached the stage of overheated vapor. The sensors exported measurements each second using a data acquisition system. Pressure drop final results are the average of the pressure drop measurements in the last 5 min of each experiment. The list of experiments analyzed in multiphase operating conditions and their results are shown in Table 2, where the mean average error (MAE) denotes the error between each measurement in every time step and the average pressure drop result.

Considering the MAE in WF pressure drop results in Table 2, a moderate error has been obtained, considering the intermittent nature of multiphase flow, enhanced by the complex geometry in the WF side of the boiler studied. Before addressing predictive tools, it is important to point out some of the anomalies that influence two-phase flow behavior and, therefore, pressure drop. As mentioned earlier, a relatively high-pressure drop in micro channels can result in significant property changes, particularly specific volume and enthalpy of the individual phases, which lead to uncertainties and instabilities in the experimental results obtained and displayed in Table 2. These results contrast with the single-phase experiments shown in Table 1, whose experimental results were straightforward to obtain in the test bench and showed quite a linear response. In multiphase experiments, sensors occasionally showed sudden offsets in the measurements of pressure drop. This phenomenon is attributed to the number of mixing chambers of the boiler, which enhances the formation of vapor pockets in these low-velocity regions. When the vapor pockets are detached from the walls of the boiler, it is possible to modify the pressure drop measurements. This issue will be tackled in future work by inducing vibrations in the boiler to prevent vapor pockets from accumulating in the mixing chamber.

## 4. Meshing

**Figure 3:**
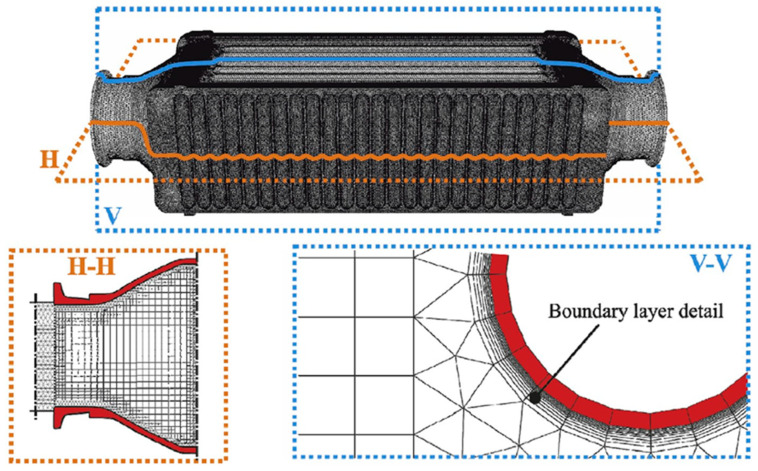
Detail of the mesh in the gas side, where H-H is a horizontal cutting plane, and V-V is a vertical cutting plane.

The surfaces of the geometry, created in CATIA v5, were discretized with triangular elements, with an average size of the edge of each element of 1 mm, whereas the volume of the elements were meshed using ANSYS Fluent. The volume of the solid tubes was created by sweeping the triangular surface elements into prisms in the normal direction of each surface. In the gas side, to better model the near wall region [80], the triangular elements in the surface were swept into 10 prisms in the normal direction of each element of the surface, with the first element having a thickness of 0.015 mm with a growth ratio of every subsequent element of 1.15, to model the boundary layer. The meshing scheme used to fill the rest of the gas volume was Hexcore, filling the gaps between hexahedral elements and prisms with tetrahedral elements. The resulting mesh for air and metal domains sums a total element of around 60,000,000 volume elements, with a maximum skewness of 0.90 and volume average skewness of 0.173. Mesh element size was limited by tube distribution, with a narrow gap of about 2.5 mm between one another. As the ANSYS Fluent manual [76] suggests, a boundary layer should be meshed in the wall to properly model the law-of-the-wall. A mesh configuration of 0.5 mm triangles was shown to be adequate for the geometry of the boiler since a coarser mesh generated many high skewness elements that could lead to instabilities or even divergence of the mesh. This mesh size and quality were already validated in a previous work [87] and did not represent a huge computational effort. A detail of the CFD mesh is displayed in Figure 3. The 1-dimensional WF mesh was created with an external routine created with the Used Defined Functions of ANSYS Fluent, which will be further explained in the following section of the present paper.

## 5. Numerical Model

**Figure 4:**
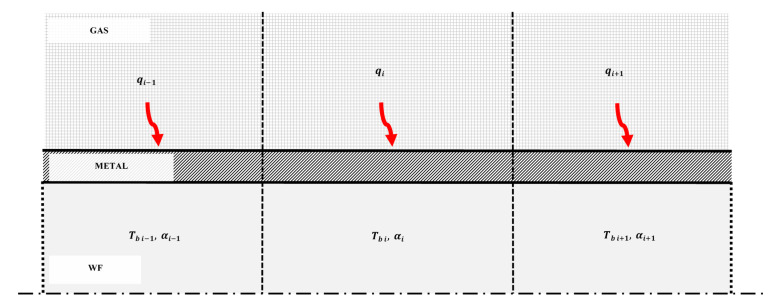
Layout and schematic paradigm of the discretized model proposed, where red arrows represent the heat flux.

The CFD software used to run the simulations of the present paper was ANSYS FLUENT 2022R2. The 1-dimensional model was programmed in an ad hoc algorithm and implemented using User Defined Functions (UDF) [88], which allows modification of their default heat transfer laws, among many other functionalities. As previously mentioned, multiphase CFD solvers have some limitations: difficulty to model heat and mass transfer mechanisms locally in many flow regimes, great computational costs, and numerical instabilities. The methodology in which this paper is based [87] was conceived as an effort to these limitations while still benefiting from the advantages of CFD software. The general approach, summarized in Figure 4, consists of the coupling of two computational domains, namely, the gas and metal side. Three-dimensional meshes are generated using the ANSYS meshing functionalities, and the WF side, where a one-dimensional mesh is generated and coupled with its metal frontier by specifically developed external algorithms.

The first step of the methodology implementation consists in discretizing the flow along the tubes in ordered cells into a 1-dimensional mesh flow along the WF path. This is achieved by programming a UDF macro called DEFINE_ON_DEMAND, which is a general-purpose macro that you can use to specify a UDF that is executed “on demand” in ANSYS Fluent, rather than having ANSYS Fluent call it automatically during the calculation. A 1-dimensional cell index is assigned to each CFD cell adjacent to the WF, based in its coordinates, and this cell index is stored using a user-defined memory. The calculations regarding this 1-dimensional mesh are not calculated by ANSYS Fluent itself, but with another UDF macro that updates the values of heat transfer coefficient and bulk temperature in each iteration by programming a DEFINE_EXECUTE_AT_END macro, which contains the physical model described in a previous work [87] and in the correlations described in Section 6 of this paper. Another function of this macro is to compute the increment of the enthalpy of each 1-dimensional cell by accounting the total energy absorbed in it through the walls adjacent to the WF fluid domain.

The DEFINE_EXECUTE_AT_END macro stores the heat transfer coefficient and bulk in another user-defined memory, and this information is transferred to ANSYS Fluent Solver using a DEFINE_PROFILE macro, which is implemented in the boundary adjacent to the WF side. This process is updated in each iteration of ANSYS Fluent solver, coupling the 1-dimensional and 3-dimensional calculations, and is repeated until there is convergence in the solution. The thermodynamic properties of the WF were obtained using NIST REFPROP.

The total WF mesh size in this simulation has 35,000 elements, with a total of 50 elements in each one of the 700 tubes. In a preliminary grid independence study, this 1-dimensional mesh size was proven to be acceptable, since finer grid elements generated oscillations in the boundary between phases and in regions with high wall heat flux gradients. On the other hand, coarser elements led to uncertainties in the onset of boiling. In any case, the 35,000 elements of the 1-dimensional mesh meant very little computational effort, and a finer mesh was shown to be a bigger concern than a coarser one.

Note that the numerical methodology described is independent from the physical model chosen. The only requirement for implementing this methodology is that the WF flow can be parametrized into a 1-dimensional path.

Regarding the particularities of this geometry, namely, those regions that cannot be easily parametrized, in the mixing chambers and the inlet and outlet manifolds, perfect mixture was assumed, averaging the enthalpies of the paths of fluids involved. Inlet enthalpies in any row of tubes that departs from one mixing chamber and converges in the following mixing chamber (referred to this paper as a row of tubes), is the mass flow average outlet enthalpy of the immediately previous row of tubes.

To model the mass flow distribution in tubes belonging to the same row, the same pressure drop was assumed for each row. Since not every tube in the same row of tubes absorbs the same amount of heat from the air and, as consequence, the WF in each tube of the same row is thermodynamically different from one another, the WF mass flow in each individual tube is modified in order to adjust the pressure drop in each individual tube to achieve a common pressure drop in every tube of the same row.

The numerical approach to estimate mass flow distribution consists in calculating, for every tube in every single row of tubes, the pressure drop of those outcomes with every mass flow possibility within a reasonable range. Having the pressure drop per tube as a function of its mass flow, the target is to find a combination of pressure drop and mass flow per tube that accomplishes the conditions of Equations (1) and (2) at the same time:

(1) $$\sum {\dot{m}}_{i}-{\dot{m}}_{T}=0$$

(2) $$\Delta p\left({\dot{m}}_{i}\right)=constant$$

where ${\dot{m}}_{i}$ stands for the mass flow of each tube of a row of tubes and ${\dot{m}}_{T}$ is the total mass flow in a column of tubes and the total mass flow of WF in the boiler; $\Delta p\left({\dot{m}}_{i}\right)$ is the targeted solution that stands for the pressure drop in a single tube that must be the same in every tube in the same row for parallel tubes, regardless of the mass flow in each tube. This equation was solved using the secant method (Equation (3)):

(3) $$\Delta p_{n+1}=\Delta p_{n}-\frac{\Delta p_{n}-\Delta p_{n-1}}{{\left(\sum {\dot{m}}_{i}-{\dot{m}}_{T}\right)}_{\Delta p_{n}}-{\left(\sum {\dot{m}}_{i}-{\dot{m}}_{T}\right)}_{\Delta p_{n-1}}}{\left(\sum {\dot{m}}_{i}-{\dot{m}}_{T}\right)}_{\Delta p_{n}}$$

Note that this will always find one solution in the single-phase stage, since the Darcy–Weisbach correlation (later discussed in Equation (4)) is linear. However, multiphase pressure drop nature is not linear, hence the secant method might find multiple solutions depending on the initial conditions, particularly in the case where some tubes started the multiphase stage and the rest remain in the subcooled liquid stage. In that case, every solution is recorded, and the lowest pressure solution of all of them is implemented in the CFD calculations. Note that the existence of multiple solutions could be translated into instabilities in the physics of the evaporation process. This scenario was not found in this study, but the methodology considers this possibility.

## 6. Pressure Drop Modeling

The general approach to obtain a predictive tool for the pressure drop in the boiler studied is to combine bibliographic correlations, experimental results, and CFD simulations. While bibliographic correlations for pressure drop are usually designed for simple and 1-dimensional geometries, the boiler studied, as already described, has several mixing chambers, which will contribute locally in the total pressure drop of the boiler. Hence, implementing only 1-dimensional bibliographic correlations in the CFD simulations will lead to an underprediction of the total pressure drop. This local pressure drop is estimated by calculating the difference between experimental pressure drop and CFD simulations using only 1-dimensional correlations.

In this section of the present paper, the proposed methodology of pressure drop modeling is broken down in two main steps: Single-phase pressure drop modeling and multiphase pressure drop modeling. Bibliographic correlation, numerical considerations, and results for local pressure drop factor are described.

### 6.1. Single-Phase Pressure Drop

**Figure 5:**
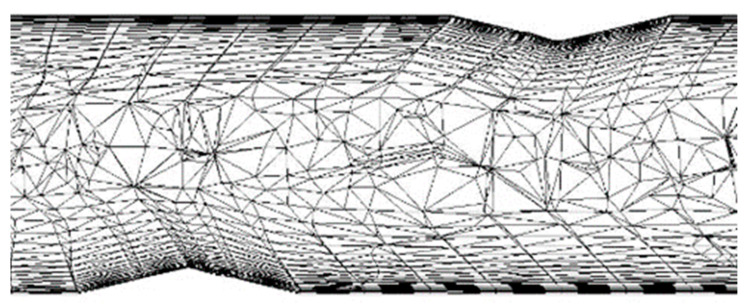
Detail of the CFD mesh of the tubes used in this part of this study.

The experiments shown in Table 1 were simulated in a full CFD single-phase calculation. The mesh described in Section 4 was modified to include a CFD 3-dimensional mesh to compute the WF domain as well. The resulting mesh has about 95 million cells, with approximately 500,000 cells each tube. A detail of the CFD of each tube is shown in Figure 5. A mesh convergence study was performed showing that the simulations reached convergence with y+ lower than 0.15 which, in the case of the meshes studied, means that the first boundary layer has a height of 0.015 mm, with 18 boundary layers and a surface mesh of triangles with an edge length of 1 mm. In this part of the study, a standard CFD simulation was launched, without using external routines.

In Table 3, the results obtained with these simulations are shown using the nomenclature of the experiments previously detailed in Table 1. Comparing these 5 experiments with the CFD results in standard simulations, a mean average error (MAE) of 9.27% was obtained. This relatively good agreement between CFD capabilities and experimental results is a good starting point to adjust the bibliographic correlations to the experiments. In addition, full CFD pressure drop results can be broken down in each geometrical component, thus the contribution on the total pressure drop of the tubes and the mixing chambers can be quantified.

The objective of the implementation in the single-phase stage of the CFD coupled with the 1-dimensional model is the Darcy–Weisbach equation [89], where f is the friction factor and the parameter to be determined in the present section of this study.

(4) $$\Delta p=\frac{f\rho v^{2}}{2}\cdot \frac{L}{D_{h}}$$

In laminar flow, the friction factor is given by the well-established Equation (5), which is a consequence of the Navier–Stokes equations.

(5) flam, sf=64Re

Note that, although Equation (5) is used for circular section tubes, the Nikuradse diagrams predict that, for a low Reynolds number (laminar flow), friction factor is not a function of relative roughness. This statement has been proven true for corrugated tubes in preliminary simulations of this study. As expected, according to the Nikuradse diagram, for turbulent flow the friction factor is higher in a corrugated tube than in the equivalent smooth tube of the same diameter and Reynolds number. As a consequence, the next step in this study is the evaluation of the friction factor in the corrugated tube studied for turbulent flow. This was obtained by accounting for the pressure drop contribution of the tubes in CFD simulations. To this regard, a series of 20 CFD simulations were launched using 20 bar liquid ethanol as WF at 80 °C, with a mass flux in the tubes in a range from $12.48\;\mathrm{kg}/m^{2}s$ to $\;449.11\;\mathrm{kg}/m^{2}s$. Taking only the simulations where the WF reached a turbulent regimen, and using a linear regression analysis, the correlation for monophasic friction factor of the corrugated tubes inside the boiler for single-phase turbulent flow ($f_{turb,sf})\;$ obtained is shown in Equation (6) as a function of the Reynolds number (Re):

(6) $$f_{turb,sf}=e^{-3.218\;{\left(lnRe\right)}^{0.2589}+3.007}$$

Once a friction factor correlation is obtained for the tubes, the next step of this study is to model the local pressure drop factor in the single-phase stage, $k_{sf}.\;$ The analytical expression for pressure drop of the WF in the boiler, including local pressure drop considerations, is shown in Equation (7):

(7) $$\Delta p=\left(\sum f\frac{L}{D}+\sum k_{sf}\right)\frac{v^{2}\rho }{2}\;$$

For the sake of simplicity, it is be assumed that this local pressure drop factor is the same for inlets and outlets where, in the case of the boiler of study, it has a total of 50 inlets in the tubes and 50 outlets from the tubes. Then, the expression for the local pressure drop factor is shown in Equation (8):

(8) $$k_{sf}=\frac{\Delta p\frac{2}{v^{2}\rho }-\sum_{i=1}^{i=tubes}f_{i}\frac{L}{D}}{n_{inlets}+n_{outlets}}$$

Observing the CFD results of the experiments from Table 3, and taking into account the laminar and turbulent friction factor in the tubes discussed in Equations (5) and (6), the single-phase local pressure drop factor $k_{sf}$ in the mixing chambers was estimated with a linear regression analysis using MATLAB. Thus, a correlation for the local pressure drop factor in single-phase flow was obtained and is shown in Equation (9), where the values of a, b, and c coefficients are shown in Table 4:

(9) $$k_{sf}=e^{a\;{\left(lnRe\right)}^{b}+c}$$

**Figure 6:**
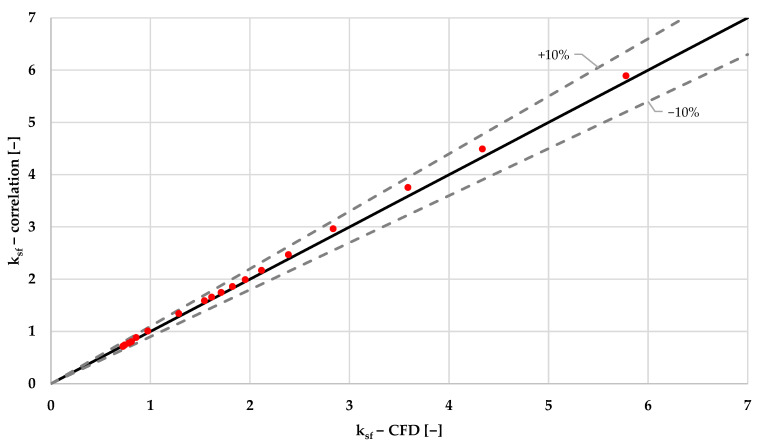
CFD vs. analytical $k_{sf}$ of local pressure drop for single-phase simulations.

The correlation described in Equation (9) and in Table 4 for single-phase local pressure drop factor shows good agreement with experimental data, as is shown in the graph of Figure 6.

### 6.2. Multiphase Pressure Drop

The multiphase stage starts when the subcooled WF absorbs enough heat to reach saturation temperature. The progressive evaporation causes axial acceleration of the flow which increases both the wall shear stress and pressure gradient along the tube. There are a number of authors that studied pressure drop in multiphase flow in mini/micro channels, as previously discussed in the introduction. In this paper, the bibliographic correlation chosen to model multiphase pressure drop was proposed by Kim and Mudawar [68]. This correlation has been obtained from a big database of different fluids in a wide range of pressures and mass fluxes, that includes the operating conditions of the boiler of study. The correlation of Kim and Mudawar for pressure drop evaporating mini/micro channels is summarized in Table 5.

For the correlation for the pressure drop, the authors suggest in their paper that in the case of dryout, the correlation is no longer valid. The same authors, Kim and Mudawar, also studied and modeled the onset of dryout in mini/micro channels [90]. They gathered a database of experiments and correlations and proposed a new one based on statistical analysis. This correlation, shown in Equation (10), was used in the present paper to determine the vapor quality for dryout incipience ($\chi_{di}$).

(10) $$\chi_{di}=1.4We_{fo}^{0.03}P_{R}^{0.08}-15.0{\left(Bo\frac{P_{H}}{P_{F}}\right)}^{0.15}Ca^{0.35}{\left(\frac{\rho_{g}}{\rho_{f}}\right)}^{0.06}$$

A dryout pressure drop correlation has not been found in the literature, so the pressure drop in this stage is obtained by blending the pressure drop for the unit of length at the point of dryout incipience and the pressure drop for the unit of length at the point of saturated vapor. The mathematical expression of this blending is described in Equations (11) and (12), where $\gamma_{dryout}$ stands for the blending factor, χ is the vapor quality, and $\;{\frac{dp}{dx}}_{go}$ and ${\frac{dp}{dx}}_{g}$ stand for the pressure drop gradient of saturated vapor and the pressure drop gradient of the vapor phase, respectively.

(11) $${\frac{dp}{dx}}_{dryout}=\gamma_{dryout}{\frac{dp}{dx}}_{go}+\left(1-\gamma_{dryout}\right){\frac{dp}{dx}}_{g}$$

(12) $$\gamma_{dryout}=\frac{1}{1-\chi_{di\;}}\left(1-\chi \right)$$

When the correlations for the pressure drop shown in Equations (1)–(12) and Table 5 are implemented in preliminary CFD simulations and compared with the experimental data displayed in Table 2, it is clear from the simulation results that the model described so far under predicts the pressure drop in the boiler in the WF side. This outcome is attributed to the dynamics of the multiphase flow in the mixing chambers, where turbulence is severely enhanced and, as consequence, the interfacial shear stress between the liquid and the vapor phases increases. Thus, the single-phase pressure drop calculated previously is not valid to predict the pressure drop as well as the local pressure drop in multiphase flow. In order to numerically model this multiphase local pressure drop, a factor $k_{mf}$ is added to the total pressure drop model (Equation (13)):

(13) $$\Delta p=\left(\overset{i=n}{\underset{i=1}{\sum }}f_{i}\frac{L}{D}\right)\frac{v_{i}^{2}\rho_{i}}{2}+\left(\overset{j=m}{\underset{j=1}{\sum }}k_{sf}{}_{j}+\overset{m=j}{\underset{j=j}{\sum }}k_{mf}{}_{j}\right)\frac{v_{j}^{2}\rho_{j}}{2}$$

where n  stands for the total number of cells of the path of the flow through the boiler, and m  stands for the total number of singularities of the path of the WF inside the boiler, with this last one accounting for all the inlets and outlets of the tubes, including mixing chambers and the inlets and outlets of the WF manifold.

To obtain the local pressure drop factor in the multiphase stage, $k_{mf}$, a linear regression study was performed using the multiphase experiments in operating conditions shown in Table 2 and another 13 experiments carried out in the experimental test bench, where WF did not reach the stage of the overheated vapor. This set of 13 experiments are not further discussed in this paper since the WF did not complete evaporation, but the results were used to feed data to estimate $k_{mf}$.

Performing a study of the linear regression with the experimental results, with a similar procedure as conducted in the single-phase flow, the local pressure drop factor in the multiphase stage $k_{mf_{j}}\;$ shown in Equation (13) needs to be corrected with the expression displayed in Equation (14), where $\chi_{j}$ is the average vapor quality in the mixing chamber, and $Re_{v}$ is the Reynolds number with the properties of saturated vapor.

(14) $$k_{mf_{j}}=\left(1-\chi_{j}\right)\cdot 3.451\cdot 10^{7}\;\cdot {\left(Re_{v}\right)}^{-1.469}$$

In the single-phase stage, $k_{mf}$ is set to 0, while $k_{sf}$ is set to 0 during the multiphase flow. The correlation of Equation (14), in the experiments analyzed, retrieved a value of $k_{mf_{j}}\;$, a maximum value in the range of about 30 to 50 in each experimental point.

## 7. Simulation Results and Discussion

**Figure 7:**
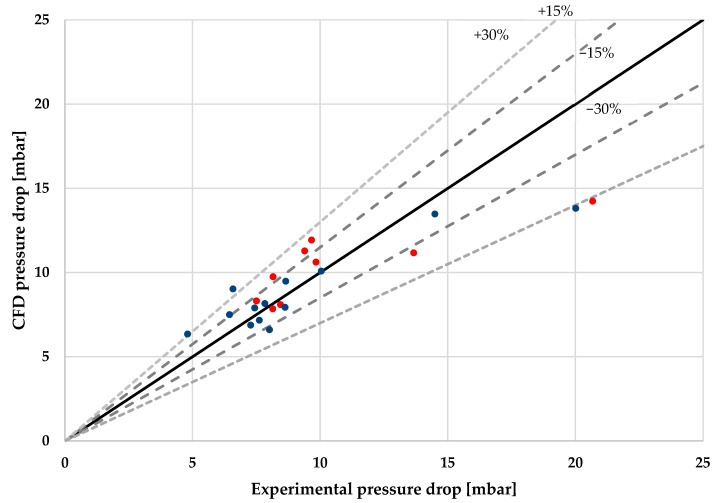
Multiphase experimental results vs. CFD on the proposed model. The red points correspond to the experiments of Table 6, while the blue points correspond to experiments where evaporation was not completed.

The overall pressure drop results of the experiments are shown in Table 2 and the physical model described in the previous section implemented in CFD simulations in ANSYS Fluent are shown in Table 6. Figure 7 graphically displays, in a dispersion graph, the 9 results of Table 6 (color red) and the 13 experimental results where evaporation was not completed, as previously mentioned. The average mean error of all 9 experimental conditions analyzed is 15.47%, with 8 of 9 multiphase experimental points inside the 30% error band, and 4 of those experiments inside the 15% error band. The experimental point outside the 30% error band is barely outside it. When the 13 data points where evaporation was not completed are included in the analysis, the average mean error is 14.57%, with 10 experimental points outside the 15% error band and 4 experiments outside the 30% error band.

**Figure 8:**
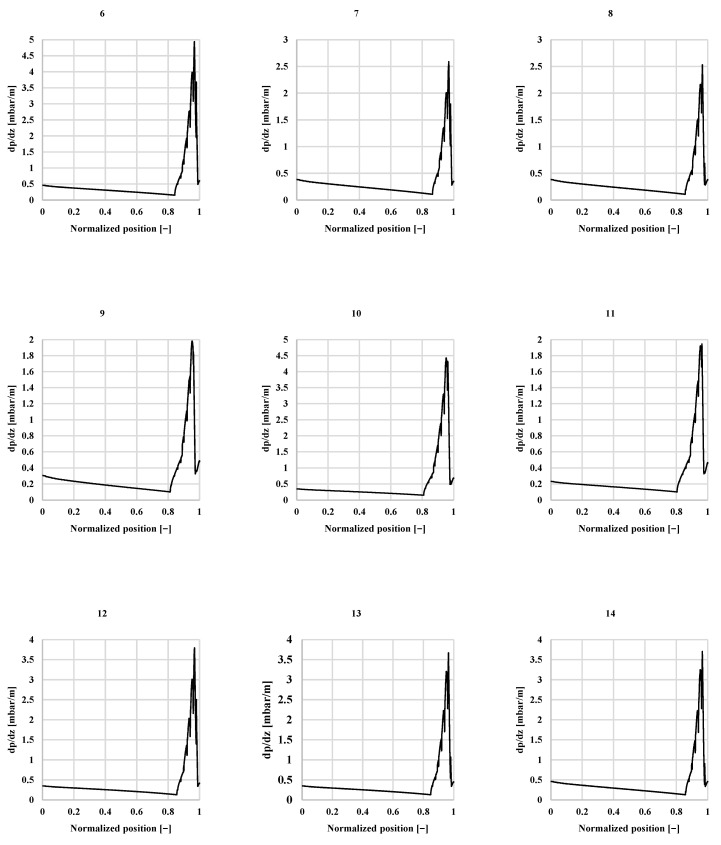
Pressure drop per unit of length of the path of the WF in the experiments analyzed, experiment codes from 6 to 14.

The methodology and correlations previously described allow to obtain an estimation of the contributions to the total pressure drop in the different features of the boiler, resulting in a distribution of pressure drop as shown in Table 7. This table suggests that the greatest impact on the total pressure drop of the boiler is mainly due to the effect of the mixing chambers on the multiphase stage, in spite of WF remaining in the multiphase stage for much shorter length than the single-phase stage (see Table 8 and Figure 8).

The distribution of the length of the path of the WF that undergoes single-phase and multiphase stages, detailed in Table 8, can be graphically depicted in the graphs in Figure 8. Those graphs represent the average pressure drop in a normalized position in each path of the WF, where position “0” stands for the WF inlet and position “1” stands for the outlet of the WF. Note that when the graphs present a high increment on the value of pressure drop gradient $\left(dp/dx\right)$, at around the position 0.8–0.85 in every experiment displayed, this means that the WF has begun the phase transition until it is quite close to the outlet of the boiler, where dp/dx is then low and stable again for a short fraction of the boiler, which represents the stage of overheated vapor. Shortly before the last single-phase stage, the WF undergoes a sharp decrease in dp/dx, which stands for the dryout stage. Only just before exiting the boiler, the dp/dx slightly increases in the stage of overheated vapor.

Note that the pressure drop in the multiphase stage is non-linear and does not follow the intuitive pattern of the Darcy–Weisbach correlation (Equation (4)), where higher mass flow rate means higher pressure drop. In general, when the multiphase flow is involved, this will not be necessarily true. As shown in Figure 8, the multiphase stage has a remarkable impact on the pressure drop of the boiler. The highest peaks of the pressure drop gradient occur in the multiphase flow and the multiphase local pressure drop factor is significantly higher than the local pressure drop factor in the single-phase stage, given by Table 6. Therefore, comparing the pressure drop of similar experiments of the boiler, it seems that the highest pressure drop is found in the experiments where the WF remains in the multiphase transition for a longer portion of the WF path, not necessarily the experiment of higher mass flow. As a consequence, and for a lesser pressure drop purpose, counter flow involves a better performance for this specific boiler. The highest temperature gradient between the fluids involved in the boiler (WF and air, in this case) is produced near the multiphase stage of the WF, meaning a higher heat exchange between fluid and, therefore, the shortest presence on the multiphase stage of the WF.

## 8. Conclusions and Future Work

This paper expands the capabilities of the work previously presented in 2019 by the same authors of the present paper, in which a methodology to calculate heat transfer in multiphase flow was developed. The contribution in the present study consists of a methodology to estimate the pressure drop in multiphase flow by means of CFD simulations, and implement it in a boiler of an ORC in a WHRS. This was accomplished by combining CFD simulations, bibliographic correlations for pressure drop, numerical analysis and experimental results, to obtain a correlation for the local pressure drop factor to the specific geometry of the boiler studied and create a tool to be implemented in the CFD software ANSYS Fluent. Besides overall pressure drop, the methodology developed also identifies the contribution of different features of the WF flow and concluded that the main contributor on the total pressure drop of the boiler is the local pressure drop on the multiphase stage. The proposed methodology also allows to predict the mass flow distribution in a bundle of parallel tubes in the single-phase stage and the multiphase stage.

Regarding the results in the pressure drop, the simulations showed an acceptable agreement with experimental results obtained in the operating conditions of the boiler, with a mean average error of 15.47%, with 8 of 9 multiphase experimental points inside the 30% error band, and 4 of those experiments inside the 15% error band.

Future work will mainly target the experimental procedure. The work will focus on expanding the database of experimental results and adjusting the proposed correlation if needed. Additionally, the methodology should be tested with different geometries with different challenges in local pressure drop assessments.

## Figures and Tables

**Table 1:** Set of experiments of monophasic flow.

	WF (Ethanol)				Air	
Experiment Code	Mass Flow (kg/h)	Inlet Temperature (°C)	Absolute Pressure (bar)	Experimental WF Pressure Drop (mbar)	Mass Flow (kg/h)	Inlet Temperature (°C)
1	64	50	2	16.36	60	100
2	73.7	50	2	20.93	60	100
3	84	50	2	25.62	60	100
4	94	50	2	30.72	60	100
5	103.1	50	2	36.33	60	100

**Table 2:** Set of multiphase experiments of the boiler in operating conditions.

	WF (Ethanol)			Air		Measurements	
Experiment Code	Mass Flow (kg/h)	Inlet Temperature (°C)	Absolute Pressure (bar)	Mass Flow (kg/h)	Inlet Temperature (°C)	WF Pressure Drop (mbar)	MAE (mbar)
6	30	60	10	64	640	13.66	0.28
7	25	60	10	60	600	7.50	0.26
8	25	60	10	61	610	8.15	0.34
9	20	60	10	56	560	8.44	0.28
10	30	80	10	65	650	20.67	0.29
11	20	80	10	55	550	8.14	0.35
12	30	80	15	64	640	9.83	0.29
13	30	80	15	65	650	9.39	0.32
14	30	60	15	66	660	9.66	0.36

**Table 3:** Experimental results for WF pressure drop shown in Table 1 with the CFD comparison.

	WF Pressure Drop Results	
Experiment Code	Experimental (mbar)	CFD (mbar)
1	16.36	15.73
2	20.93	19.12
3	25.62	23.15
4	30.72	27.35
5	36.33	31.52

**Table 4:** Table of coefficients of Equation (9).

	*Re* < 4000	*Re* > 4000
*a*	−394.7	7767
*b*	0.0077	−4.918
*c*	401	−0.4066

**Table 5:** Correlations for pressure drop in boiling mini/micro channels [68].

${\left(\frac{dp}{dz}\right)}_{F}={\left(\frac{dp}{dz}\right)}_{f}\Phi_{f}^{2}$	Where $\Phi_{f}^{2}=1+\frac{C}{X}+\frac{C}{X^{2}},\;X^{2}=\frac{{\left(dp/dz\right)}_{f}}{{\left(dp/dz\right)}_{g}}$
$-{\left(\frac{dp}{dz}\right)}_{f}=\frac{2f_{f}v_{f}G^{2}{\left(1-\chi \right)}^{2}}{D_{h}},-{\left(\frac{dp}{dz}\right)}_{g}=\frac{2f_{g}v_{g}G^{2}\chi^{2}}{D_{h}}$	Where $\left\{\begin{array}{c} f_{k}=16Re_{k}^{-1}\;for\;Re_{k}<2000\\ f_{k}=0.079Re_{k}^{-0.25}\;for\;2000\leq Re_{k}<20,000\\ f_{k}=0.046Re_{k}^{-0.2}\;for\;Re_{k}>2000 \end{array}\right.$
$Re_{f}\geq 2000,\;Re_{g}\geq 2000\;\left(tt\right)$	$C_{non-boiling}=0.39Re_{fo}^{0.03}Su_{go}^{0.1}{\left(\frac{\rho_{f}}{\rho_{g}}\right)}^{0.35}$
$Re_{f}\geq 2000,\;Re_{g}<2000\;\left(tv\right)$	$C_{non-boiling}=8.7\cdot 10^{-4}Re_{fo}^{0.17}Su_{go}^{0.5}{\left(\frac{\rho_{f}}{\rho_{g}}\right)}^{0.14}$
$Re_{f}<2000,\;Re_{g}\geq 2000\;\left(vt\right)$	$C_{non-boiling}=0.0015Re_{fo}^{0.59}Su_{go}^{0.19}{\left(\frac{\rho_{f}}{\rho_{g}}\right)}^{0.36}$
$Re_{f}<2000,\;Re_{g}<2000\;\left(vv\right)$	$C_{non-boiling}=3.5\cdot 10^{-5}Re_{fo}^{0.44}Su_{go}^{0.5}{\left(\frac{\rho_{f}}{\rho_{g}}\right)}^{0.48}$
$Re_{f}\geq 2000$	$C=C_{non-boiling}\left[1+60We_{fo}^{0.32}{\left(Bo\frac{P_{H}}{P_{F}}\right)}^{0.78}\right]$
$Re_{f}<2000$	$C=C_{non-boiling}\left[1+530We_{fo}^{0.52}{\left(Bo\frac{P_{H}}{P_{F}}\right)}^{1.09}\right]$

**Table 6:** Experimental and CFD pressure drop results with relative error.

Experiment Code	Experimental WF Pressure Drop (mbar)	CFD WF Pressure Drop (mbar)	Relative Error (%)
6	13.66	11.17	18.20%
7	7.50	8.32	10.88%
8	8.15	9.75	19.68%
9	8.44	8.10	4.04%
10	20.67	14.24	31.10%
11	8.14	7.85	3.57%
12	9.83	10.62	8.03%
13	9.39	11.29	20.19%
14	9.66	11.93	23.54%

**Table 7:** Pressure drop contributions.

	Single Phase		Multiphase	
Experiment Code	Tubes	Mixing Chamber	Tubes	Mixing Chamber
6	18.74%	17.29%	15.71%	48.26%
7	11.72%	18.56%	16.04%	53.68%
8	10.00%	15.67%	13.34%	60.99%
9	12.23%	15.06%	15.65%	57.07%
10	16.01%	11.36%	10.92%	61.71%
11	13.21%	13.45%	15.26%	58.08%
12	13.91%	14.95%	15.20%	55.95%
13	13.13%	14.01%	14.23%	58.63%
14	11.77%	15.63%	14.41%	58.19%

**Table 8:** Percentage of length in the single-phase stage and multiphase stage.

Experiment Code	Single Phase	Multiphase
6	85.27%	14.73%
7	87.71%	12.29%
8	87.19%	12.81%
9	83.90%	16.10%
10	82.83%	17.17%
11	82.70%	17.30%
12	86.66%	13.34%
13	86.34%	13.66%
14	87.37%	12.63%

## Data Availability

Not applicable.
